# Absorption behavior of riociguat: bioavailability, food effects, and dose-proportionality

**DOI:** 10.1186/2050-6511-14-S1-P7

**Published:** 2013-08-29

**Authors:** Corina Becker, Reiner Frey, Christiane Hesse, Sigrun Unger, Michael Reber, Wolfgang Mueck

**Affiliations:** 1 Clinical Pharmacology, Bayer HealthCare Pharmaceuticals, Wuppertal, Germany; 2 Global Biostatistics, Bayer HealthCare Pharmaceuticals, Wuppertal, Germany

## Background

Riociguat, an oral soluble guanylate cyclase (sGC) stimulator, is currently being investigated for the treatment of pulmonary hypertension. Riociguat has a novel dual mode of action, directly stimulating sGC, independent of nitric oxide (NO), and increasing sensitivity of sGC to NO. Riociguat thereby restores the NO–sGC–cGMP pathway, which is impaired in pulmonary hypertension. Three pharmacokinetic studies were performed to characterize the absorption behavior of riociguat including absolute bioavailability, food effects, and dose-proportionality.

## Methods

The pharmacokinetic and safety profiles of riociguat were investigated in three open-label, randomized, crossover studies in healthy male subjects. In the absolute bioavailability study, fasted subjects (n=22) received a single oral immediate-release dose of riociguat 1 mg or intravenous riociguat 1 mg. In the food-effects study, subjects (n=23) received a single oral dose of immediate-release riociguat 2.5 mg after a 10-hour fast or after a high-fat high-calorie breakfast eaten within 30 minutes of dosing. In the dose-proportionality study, fasted subjects (n=24) received a single oral dose of riociguat 0.5, 1, 1.5, 2, or 2.5 mg.

## Results

**Figure 1:**
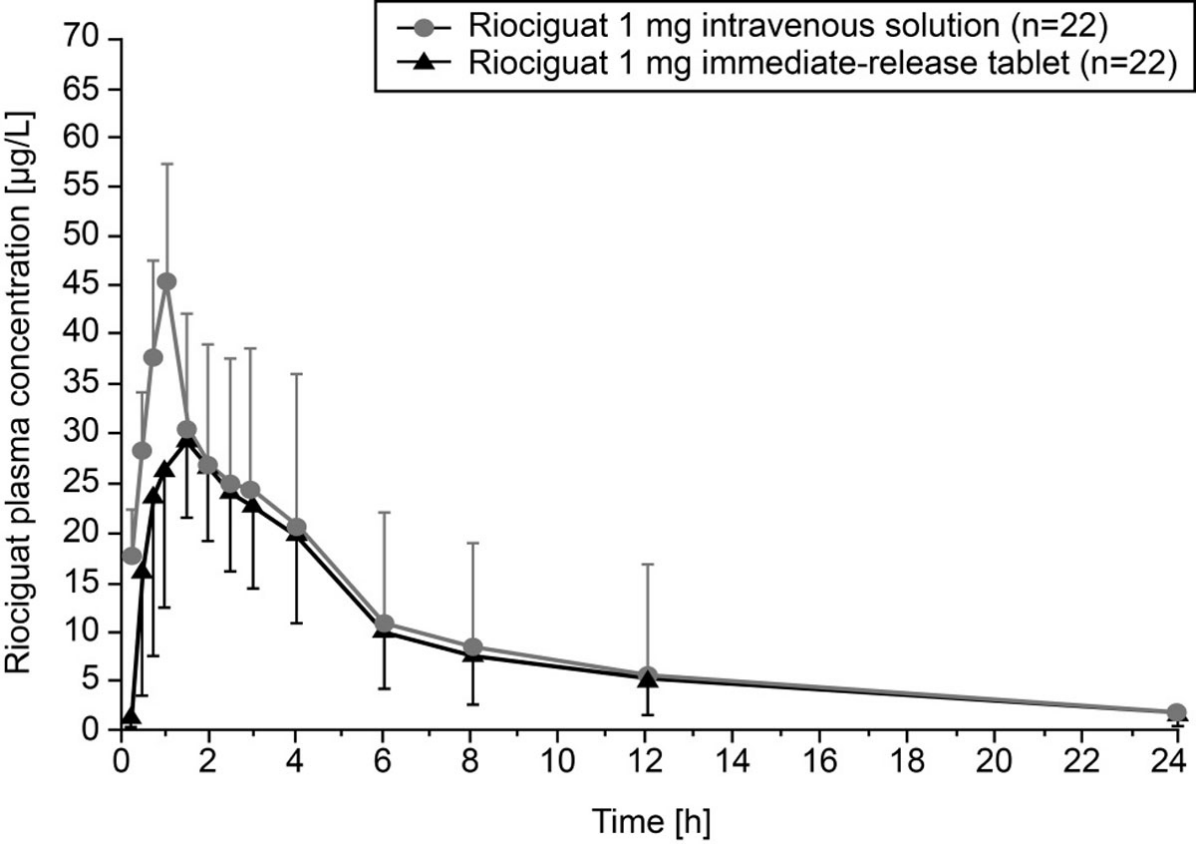
Plasma riociguat concentrations following single 1 mg intravenous (n=22) or oral doses of riociguat (n=22) (absolute bioavailability study).

**Figure 2:**
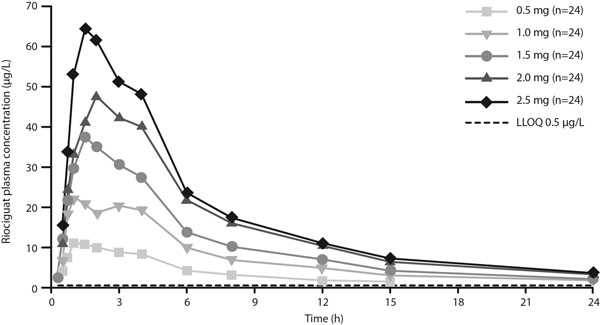
Mean plasma riociguat concentrations following single oral doses of riociguat 0.5, 1.0, 1.5, 2.0, or 2.5 mg (n=24) (dose-proportionality study). LLOQ, lower limit of quantification.

In the absolute bioavailability study, riociguat exposure was similar after oral and intravenous dosing and oral bioavailability was 94% (95% confidence interval: 83–107) (Figure 1). Mean C_max_ of riociguat was slightly lower after oral dosing compared with intravenous dosing. In the food-effects study, a high-fat breakfast had little effect on the extent of riociguat absorption, although absorption was delayed (Table 1). In fed subjects, the C_max_ of riociguat decreased by 35.3% and t_max_ increased compared with fasted subjects. In the dose-proportionality study, systemic exposure of riociguat was dose-proportional over 0.5–2.5 mg (Figure 2) with low intra-individual variability and moderate-to-high inter-individual variability. Riociguat was well tolerated in all studies. The most common treatment-emergent and riociguat-related adverse events were headache, flushing, and nasal congestion across all studies.

**Table 1:** Riociguat pharmacokinetics following a single oral dose of riociguat 2.5 mg in fed and fasted subjects (food-effects study)

Parameter^a^	Fasted (n=23)	Fed (n=23)	Estimated fed:fasted ratio (%)	90% CI	%CV
C_max_ (μg/L)	84.2 (44.7−152.7)	54.8 (28.9−91.4)	64.7	57.8–72.5	22.5
t_max_ (h)	1 (0.5−4.0)	4 (1.5−6.0)	—	—	—
AUC_0-∞_ (μg⋅h/L)	572.2 (112.3−1300.0)	505.6 (113.3−1205.0)	88.3	82.2–95.0	14.3

AUC, area under the plasma concentration–time curve; CI, confidence interval; C_max_, maximum riociguat plasma concentration; CV, coefficient of variation; t_max_, time to reach C_max_.

^a^Data are mean (range) except t_max_, which is median (range).

## Conclusion

Riociguat shows complete oral absorption with no clinically relevant food effects; riociguat can therefore be taken with or without food. Riociguat systemic exposure increased dose proportionally over all doses (0.5–2.5 mg), supporting the suitability of the individualized dose-titration scheme used in the Phase III pulmonary arterial hypertension (PATENT) and chronic thromboembolic pulmonary hypertension (CHEST) studies.

